# Application-based remote interstage home monitoring for infants with shunt- or duct-dependent pulmonary perfusion

**DOI:** 10.3389/fcvm.2024.1493698

**Published:** 2025-01-06

**Authors:** Lisa-Maria Rosenthal, Friederike Danne, Sophie de Belsunce, Lisa Spath, Chiara-Aiyleen Badur, Joachim Photiadis, Felix Berger, Katharina Schmitt

**Affiliations:** ^1^ Department of Congenital Heart Disease—Pediatric Cardiology, Deutsches Herzzentrum der Charité (DHZC), Berlin, Germany; ^2^ Berlin Institute of Health, Berlin, Germany; ^3^ German Centre for Cardiovascular Research, Partner Site Berlin, Berlin, Germany; ^4^ Department of Congenital Heart Surgery, Deutsches Herzzentrum der Charité (DHZC), Berlin, Germany; ^5^ Department of Developmental Pediatrics, Deutsches Herzzentrum der Charité (DHZC), Berlin, Germany

**Keywords:** remote patient monitoring, interstage monitoring, application-based monitoring, single ventricle heart disease, Norwood palliation, shunt-dependent pulmonary perfusion, duct-dependent pulmonary perfusion

## Abstract

**Objective:**

Interstage home monitoring (IHM) programs are considered standard of care after Norwood palliation and have led to substantial improvements in clinical outcomes. This study aims to evaluate an application-based remote IHM program for infants with shunt- or duct-dependent pulmonary circulation. The primary goals were to discharge infants from the hospital while minimizing mortality, optimizing somatic growth, and enhancing caregivers' confidence in the clinical management at home.

**Methods:**

Infants with shunt-dependent single ventricle physiology or complex biventricular physiology requiring staged palliation with aortopulmonary shunt were enrolled for the study. Caregivers completed a comprehensive education program on the clinical management of their child at home and were asked to remotely send monitoring data using an application. We analyzed demographic data and clinical outcomes; evaluated patient acceptance and adherence, as well as data entry patterns and metrics; and compared these to a historical control group monitored in a non-remote IHM program and with a propensity score-matched cohort adjusted for baseline characteristics.

**Results:**

We enrolled 30 infants in the remote IHM program between July 2021 and May 2024. The median duration of IHM was 110 days (IQR 75–140). A median of 353 (IQR 351–743) data entries were sent per patient during IHM of which 0.8% (IQR 0.3–1.9) were pathological. Readmissions (63%) and interventions (57%) were common, mainly due to cyanosis and infections. As all infants survived stage II palliation, interstage mortality could be reduced to 0% compared to 10.3% in the historical control group and was significantly lower compared to the propensity score-matched cohort with 14% (*P* = 0.032).

**Conclusion:**

Application-based remote IHM for infants with duct- or shunt-dependent pulmonary perfusion is feasible, with high acceptance and adherence. The program significantly reduced interstage mortality compared to traditional monitoring methods. Remote patient monitoring (RPM) improves communication between caregivers and healthcare teams, allowing for early intervention and optimized patient outcomes. RPM has the potential to improve outcomes, enhance patient safety, and reduce family burden in this high-risk population.

## 1 Introduction

Infants born with single ventricle heart defects (SVHD) have been experiencing substantial improvements from compassionate care to long-term survival. After Norwood palliation, physiological challenges regarding the parallel circulation with shunt-dependent pulmonary blood flow put infants at risk for life-threatening events. Infants that are considered stable may unexpectedly and suddenly deteriorate or die at home. Efforts to identify risk factors or specific targets have failed as interstage mortality has most likely multiple causes that affect the critical balance of pulmonary to systemic blood flow. Close monitoring of the vital signs, body weight, and overall clinical status is crucial for the early detection of potential problems (1). Historically, frequent clinic visits and hospitalizations were required, which can be burdensome for infants and their caregivers. Before the implementation of in-home surveillance, strategies reported mortality rates as high as 15%–18% (1–3). Nowadays interstage home monitoring (IHM) programs are considered standard of care according to current guidelines and have led to substantial improvements (4, 5). IHM mortality rates vary significantly between centers today. Large multicentered trials such as the National Pediatric Cardiology Quality Improvement Collaborative reported mortality rates between 8% and 10% in 1,050 patients treated in 52 centers after implementation of IHM, but interstage mortality improved to 5.3% during the observation period (6). The multicenter single ventricle reconstruction trial reported an interstage mortality of 12% in 426 patients who were released after stage I palliation (S1P) (7). Remote patient monitoring (RPM) has emerged as a valuable tool in the management of various diseases. RPM offers a promising solution to gather key physiological parameters from the comfort of the family's home and track the health status of children during IHM as devices such as smartphones and tablets are ubiquitously available. To date, only a few studies have evaluated the benefit of asynchronous transfer of monitoring data with tablet- or application-based remote IHM programs (8–11). We established an application-based remote patient interstage monitoring for infants with single ventricle heart disease after Norwood palliation and evaluated our initial experience regarding feasibility, acceptance, and barriers. Our goals were to minimize hospitalization, mortality, and adverse outcomes, optimize somatic growth, and increase caregivers’ confidence regarding clinical management at home.

## 2 Methods

### 2.1 Operative techniques

The Norwood procedure was performed without circulatory arrest in moderate hypothermia (28°C–32°C) using cardiopulmonary bypass with regional perfusion technique and antegrade cerebral perfusion. Enlargement of the aortic arch was performed using a pulmonary homograft patch. For patients who received an RVPA conduit (Sano shunt), a 5 or 6 mm ringed Gore-Tex prosthesis was used. For modified Blalock–Taussig shunt (mBTS), patients received a non-ringed Gore-Tex prosthesis of 3–4 mm according to body weight at the time of the surgery. Delayed sternal closure was performed in all patients.

### 2.2 Development of the app “Evie by DHZB”

The development of the application “Evie by DHZB” that was used in this trial was performed by a cross-functional team of medical staff, project managers, designers, and developers using design thinking methods. To generate first patterns and implications for the future application, parents of children with SVHD during IHM or still hospitalized after S1P were asked to test different prototypes. Video interviews were conducted with stakeholders to analyze their user experience. A minimal viable product was defined in consideration of feasibility taking into account data protection and legal requirements. The application was coded using Flutter, an open-source UI software development kit that allows the development of cross-platform applications from a single codebase (in our case Android and iOS). Data storage is on the device only and can be shared via coded e-mail with the IHM team. The application was used for research purposes only, and approval was given after a data security review by the ethical review board of Charité—Universitätsmedizin Berlin.

### 2.3 Study design

**Figure 1:**
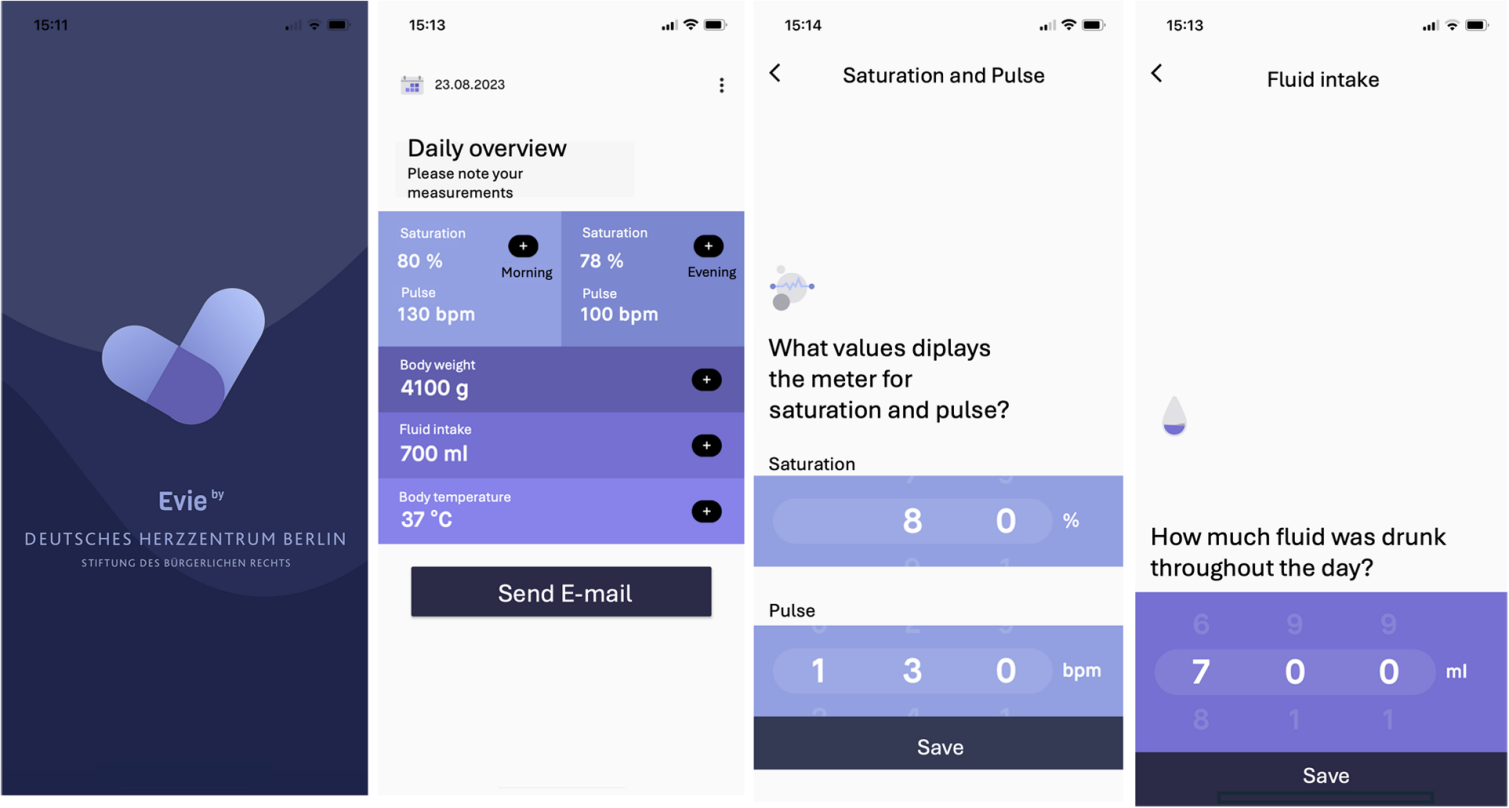
Screenshots of the application “Evie” illustrating the dashboard and data entry modalities.

Demographic and clinical data were extracted from the electronic health record. Our IHM program includes all infants with shunt-dependent single ventricle physiology or infants with complex biventricular physiology requiring staged palliation with aortopulmonary shunt. The interstage period was defined as discharge from stage I palliation (S1P or first procedure) and admission for stage II palliation (S2P) or biventricular (BV) correction (referred to as second procedure). Accordingly, interstage mortality was defined as the death of patients who were discharged after the first procedure and died before the second procedure. Care providers were provided with home monitoring equipment (monitor with pulse oximeter, scale). Parents of infants requiring IHM completed a comprehensive education program regarding the clinical management at home, red flags, and emergency situations before discharge. “Red flags” included low oxygen saturation (<70%), any vomiting or diarrhea, feeding difficulty or concerns of dehydration, weight loss over 3 days, symptoms of congestion (such as tachypnea/dyspnea, sweating, edema), irritability, or any clinical aspect that seemed worrisome to the parents. After informed consent, parents downloaded the application “Evie” (open-access, accessible for devices with Android and iOS) on their mobile phones and were asked to daily enter and send monitoring data. In the absence of access to a device, parents were provided with a tablet. The application provides a dashboard to enter clinical parameters such as oxygen saturation and heart rate (twice daily in the morning and evening), body weight, enteral intake, and body temperature (Figure 1). A comment function allows caregivers to give a subjective perspective on how their child is doing and questions can be addressed to the IHM team. Monitoring data are sent to the IHM team on a daily base. Push-up notifications remind caregivers to perform monitoring and send information to the IHM team. As data transmission failures were not monitored, parents were contacted when no data were sent for 3 days. We analyzed demographic data and clinical outcomes (interstage mortality, adverse outcomes, unplanned readmissions and interventions, somatic growth) and evaluated acceptance and caregivers’ adherence. Additionally, data entry patterns and metrics were analyzed. We compared the results with a control group of patients who underwent palliation within the 5 years before the trial and those who did not participate during the study period. Patients of the control group with single ventricle heart disease were monitored in a non-remote IHM program. They were equipped with a pulse oximeter and a weight scale, and parents received the same training before discharge as parents of the remote IHM study group. They were asked to have twice-weekly telephone contact with the IHM team to report on their infant's vital parameters and clinical state.

### 2.4 Statistical analysis

Categorial variables were described as absolute numbers with percentages, and a two-tailed Chi-square test was performed for analysis. Data distribution was visualized by histogram and normality was tested with the Kolmogorov–Smirnov test. All variables displayed non-normal distribution. Continuous variables are presented as median with interquartile ranges (IQR). Non-parametric Mann–Whitney *U*-test was used for the analysis of unpaired continuous variables and the Wilcoxon matched-pairs signed-rank test for repeated measurements of continuous variables. Multivariable Cox regression analysis was conducted to assess the risk-adjusted impact of potentially confounding variables that have been previously described to affect interstage mortality. Predictors were handled without transformation. Propensity score matching (PSM) was employed to match study patients with control cases ensuring similarity across baseline characteristics including sex, diagnosis, type or surgery at the first procedure, and shunt type. Matching was done with a caliper setting of 0.15 of the standard deviation of the logit propensity score. Within the propensity score-matched cohort, clinical and survival outcomes were compared. A *P*-value ≤ 0.05 was considered statistically significant. Data were analyzed and plotted using SPSS statistics (version 23, IBM Corp., Armonk, NY, USA) and Prism GraphPad 9 (GraphPad Software Inc., La Jolla, CA, USA).

## 3 Results

### 3.1 Baseline demographics of the study cohort

Our IHM program included all infants with shunt- or duct-dependent single ventricle physiology or infants with complex biventricular physiology requiring staged palliation with aortopulmonary shunt. Only patients who were discharged after the initial procedure were included in the analysis. In total, we included 30 patients in the remote interstage home monitoring between July 2021 and May 2024. Demographic data and surgical interventions prior to IHM are summarized in Table 1. Fifty percent of the patients were diagnosed with hypoplastic left heart syndrome (HLHS, *n* = 15), and the other patients had shunt-dependent single ventricle physiology (*n* = 12) or complex biventricular physiology (*n* = 3) requiring staged palliation with aortopulmonary shunt. The most common shunt type was a Sano shunt in 53% of all patients followed by a modified Blalock–Taussig shunt (mBTS) in 30%. Twenty-seven patients (90%) received univentricular palliation with bidirectional Glenn anastomosis or comprehensive stage II after completing IHM whereas three patients (10%) received biventricular correction. Medication at discharge is listed in Table 1. All patients were treated with aspirin to prevent shunt thrombosis. Aspirin non-responders were identified via Multiplate Analyzer. Most patients received ACE inhibitors and beta-blockers for congestive therapy. Seventeen patients (85%) were discharged with diuretics. Patients with a hemoglobin level below 16 g/dl were treated with erythropoietin. Iron supplementation was given when indicated by low transferrin saturation. Two patients experienced postoperative arrhythmia (both with Sano shunt) and were treated with amiodarone or propafenone. Two patients were treated with ivabradine for tachycardia which was not sufficiently treatable with beta-blockers.

**Table 1:** Baseline characteristics of the study patients.

Total *N* = 30	Median (IQR), *N* (%)
Age at the start of IHM (day)	48 (33–67)
Sex	
– Male	14 (47%)
– Female	16 (53%)
Diagnoses	
– HLHS	15 (50%)
– Other	15 (50%)
Other	
– AVSD	4 (13%)
– CCTGA	3 (10%)
– DORV	2 (7%)
– PA/IVS	2 (7%)
– PA/VSD	1 (3%)
– HLHC	1 (3%)
– Tricuspid atresia	1 (3%)
– Shone	1 (3%)
Dominant single ventricle	
– Right ventricle	23 (77%)
– Left ventricle	7 (23%)
Surgical interventions prior to interstage	
– Norwood	19 (63%)
– Shunt	9 (30%)
– PDA stenting	2 (7%)
– Pulmonary arterial banding	4 (13%)
Shunt type	
– Sano shunt	16 (53%)
– Modified Blalock–Taussig shunt (mBTS)	9 (30%)
– Central aortopulmonary shunt	3 (10%)
– PDA with stent	2 (7%)
Procedure after IHM	
– Bidirectional Glenn anastomosis or comprehensive stage II	27 (90%)
– Biventricular correction	3 (10%)
Cardiac medication during IHM	
– Aspirin	30 (100%)
– Beta-blockers	29 (97%)
– ACE inhibitors	27 (90%)
– Diuretics	27 (90%)
– Erythropoietin	16 (55%)
– Iron	15 (52%)
– Ivabradine	2 (7%)
– Amiodarone	1 (3%)
– Propafenone	1 (3%)

More than one option may apply to one patient.

### 3.2 Home monitoring data

**Figure 2:**
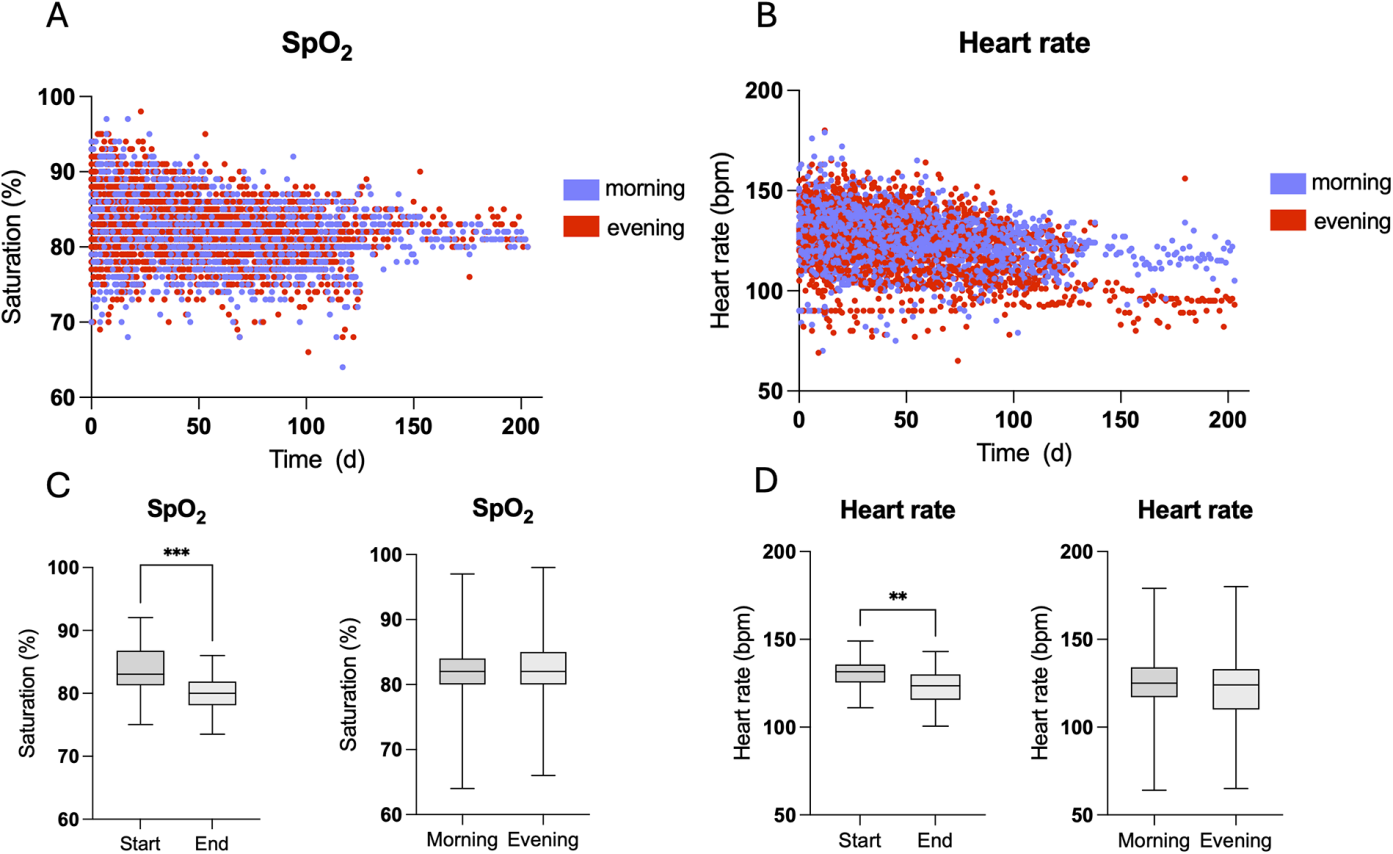
Scatter plots visualizing peripheral oxygen saturation **(**SpO_2_, **A)** and heart rate **(B)** that were measured twice daily. Both SpO_2_ **(C)** and heart rate **(D)** significantly decreased from the start to the end of interstage monitoring (IHM), whereas no differences were found between the morning and evening measurements.

**Figure 3:**
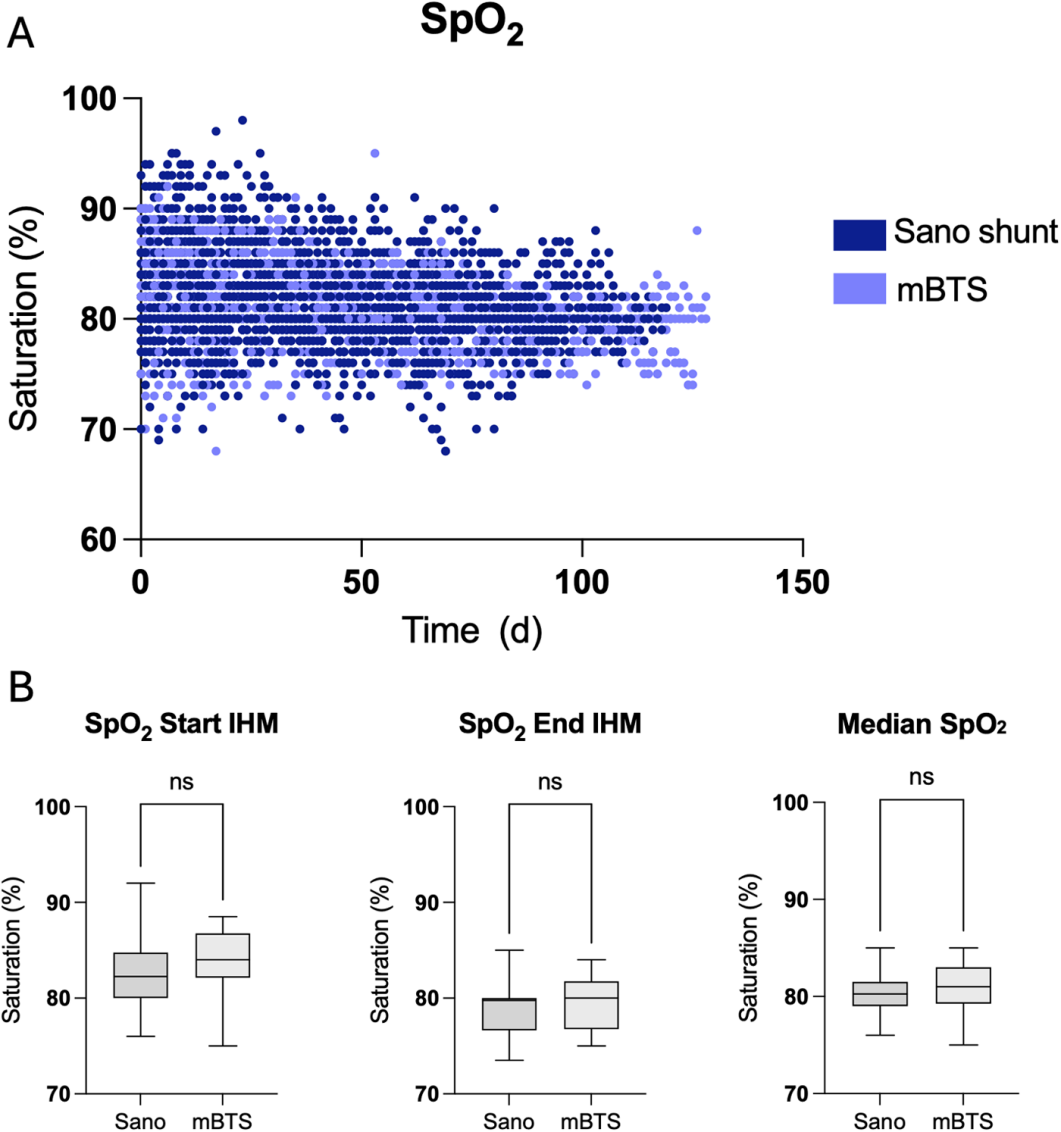
Scatter plot of peripheral oxygen saturation **(**SpO_2_, **A)** in children with either Sano shunt or modified Blalock–Taussig shunt (mBTS). SpO_2_ was higher in patients with mBTS at all time points **(B)** without reaching statistical significance.

Home monitoring data are summarized in Table 2. The duration of interstage monitoring was defined as the period from hospital discharge to readmission for the subsequent stage II palliation or biventricular correction. The median duration of IHM was 110 days (IQR 75–140). For each patient, 535 data entries (IQR 351–743) were sent during the observation period. Of all sent data entries, 0.8% (IQR 0.3–1.9) were pathological defined as peripheral oxygen saturation (SpO_2_) below 75% or above 90%, heart rate less than 75/min or greater than 170/min (for an infant that is not in distress or crying), and body temperature of 38°C or higher or failure to gain weight over 3 days. SpO_2_ and heart rate of all patients during the observation period are plotted in Figure 2. Median SpO_2_ was 81 (IQR 80–83) with a range from 68% to 98%. SpO_2_ and heart rate significantly decreased over the observation period (Figure 2). SpO_2_ and heart rate of patients with either Sano shunt or modified Blalock–Taussig shunt (mBTS) are plotted in Figure 3. SpO_2_ was slightly higher in patients with mBTS, although the difference was not statistically significant.

**Table 2:** Home monitoring data.

	Median (IQR), *N* (%)
Duration of interstage monitoring (day)	110 (75–140)
Data entry per patient	
– Total data entries (*n*)	535 (351–743)
– Missed data entries (*n*)	109 (23–237)
– Missed data entries (%)	15.6 (3.6–33.8)
– pathological entries (*n*)	4.5 (2–9)
– Pathological entries (%)	0.8 (0.3–1.9)
SpO_2_ (%)	
– Median (IQR)	81 (80–83)
– Min/max, range	64/98, 34
– Mean change across the observation window	−3 (−5.8 to 0)
HR (bpm)	
– Median (IQR)	125 (119–130)
– Min/max, range	64/180, 116
– Mean change across the observation window	−4.5 (−12.5 to 0)

### 3.3 Somatic growth

**Figure 4:**
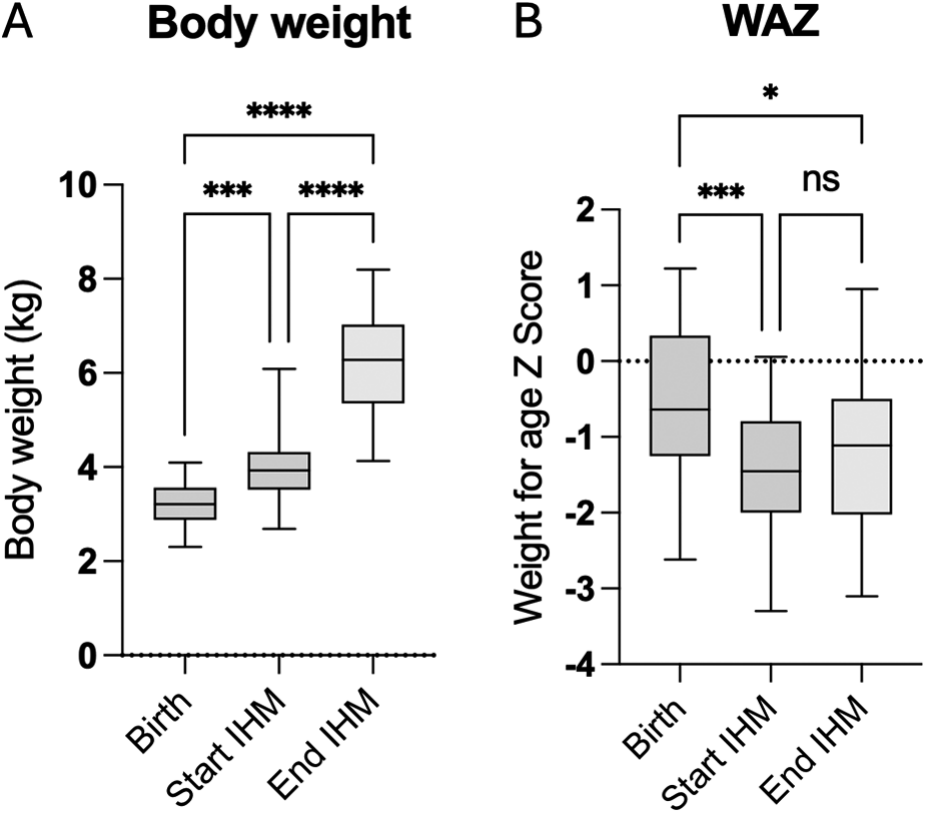
Body weight **(A)** and weight for age *Z* score **(**WAZ, **B)** at birth, at the start, and at the end of IHM surveillance.

Eighteen infants (60%) were bottle-fed at discharge, six infants (20%) required a nasogastric tube, and six infants (20%) were breastfed. Fourteen of 20 children (70%) received breast milk, and six children (39%) received formula. Sixteen patients (80%) were given a fortifier to ensure adequate caloric intake while limiting volume intake. Three infants were born with low birth weight defined as below the 10th percentile. Median body weight at the start of IHM was 3.92 kg (IQR 3.63–4.35) and increased to 6.40 kg (IQR 5.12–7.13) at the end of IHM (Figure 4). All infants successfully gained weight as body weight during the IHM. The weight for age *Z* score (WAZ) decreased significantly from birth to the start of IHM and was still significantly lower at the end of IHM than at birth. The WAZ of infants who did not manage to gain an average of 15 grams per day over the IHM period at admission for stage II surgery was significantly lower (WAZ −2.3) compared to those who gained 15 g/day (WAZ −0.95, *P* = 0.014). Of these infants, 50% were fed at least partially through a nasogastric tube compared to 9% of the infants without failure to gain an average of at least 15 g per day. This subpopulation (average weight gain < 15 g per day and nasogastric tube) had the lowest WAZ at admission to stage II with −2.63 (IQR −2.77 to −2.58). For infants without a nasogastric tube but failure to gain an average of at least 15 g per day, the median WAZ was −1.7, (IQR −2.1 to −1.1), and for infants with an average weight gain of 15 g per day, the median WAZ was −0.95 (IQR −1.6 to 0.47).

### 3.4 Adverse events and survival to stage II

Data on unplanned hospital admissions and unplanned interventions by diagnosis are listed in Table 3. During the observation period, we observed 32 unplanned admissions in the whole study group. Nineteen patients (63%) had at least one unplanned admission. The most common reasons were either cyanosis or infection. Infants admitted with cyanosis had a significantly lower oxygen saturation (mean 70.3%, IQR 68–78) in RPM within 24 h before admission when compared to the mean saturation in RPM interstage monitoring (79.4%, IQR 76%–81%, *P* = 0.0338). Mean oxygen saturation within 3 days before admission in RPM was lower (mean 77%, IQR 74–79) but not statistically different from the mean saturation during RPM. We observed 32 unplanned interventions or procedures, and 17 patients (57%) required at least one unplanned intervention. Two infants presented with hypoxic shock due to acute shunt stenosis which was immediately treated with emergency catheter-based balloon angioplasty of the shunt and concurrent stent implantation. Infants with HLHS had more unplanned hospital admissions and unplanned interventions (in total and per patient) compared to infants with other diagnoses than HLHS. The difference was not statistically significant. Three infants were switched from interstage home monitoring to in-hospital monitoring during the observation period: one infant because of cyanosis that was not sufficiently treatable, one infant because of a shunt infection/endocarditis, and one infant because of socially disadvantaged circumstances of the family. All infants that were monitored in this prospective trial survived stage II palliation or biventricular correction. Interstage mortality was 0% in this study cohort.

**Table 3:** Adverse events in the study group.

	Median (IQR), *N* (%)		
	All patients (*N* = 30)	HLHS (*N* = 15)	Other (*N* = 15)
Unplanned hospital admissions			
– Total	32	21	11
– Per patient	1 (0–2)	1 (0–2)	1 (0–1)
– Patients with at least one unplanned admission	19 (63%)	11 (73%)	8 (53%)
Indications for hospitalization^a^			
– Cyanosis	16 (53%)	10 (45%)	6 (50%)
– Infection	15 (50%)	10 (45%)	5 (42%)
– Other	3 (10%)	2 (9%)	1 (8%)
Unplanned interventions/procedures			
– Total	32	17	15
– Per patient	1 (0.5–1)	1 (0–2)	1 (0–1)
– Patients with at least one unplanned intervention/procedure	17 (57%)	9 (60%)	8 (53%)
Interventions/procedures^a^			
– Catheter examination with intervention	13^a^	8	5
Balloon angioplasty shunt/PDA stent	10	6	4
Balloon angioplasty pulmonary arteries	6	3	3
Balloon angioplasty coarctation	2	0	2
– Other (e.g., RBC transfusion, i.v. antibiotics)	6	2	4

^a^ More than one option may apply to one patient.

### 3.5 Comparison of the study cohort to a control cohort

**Figure 5:**
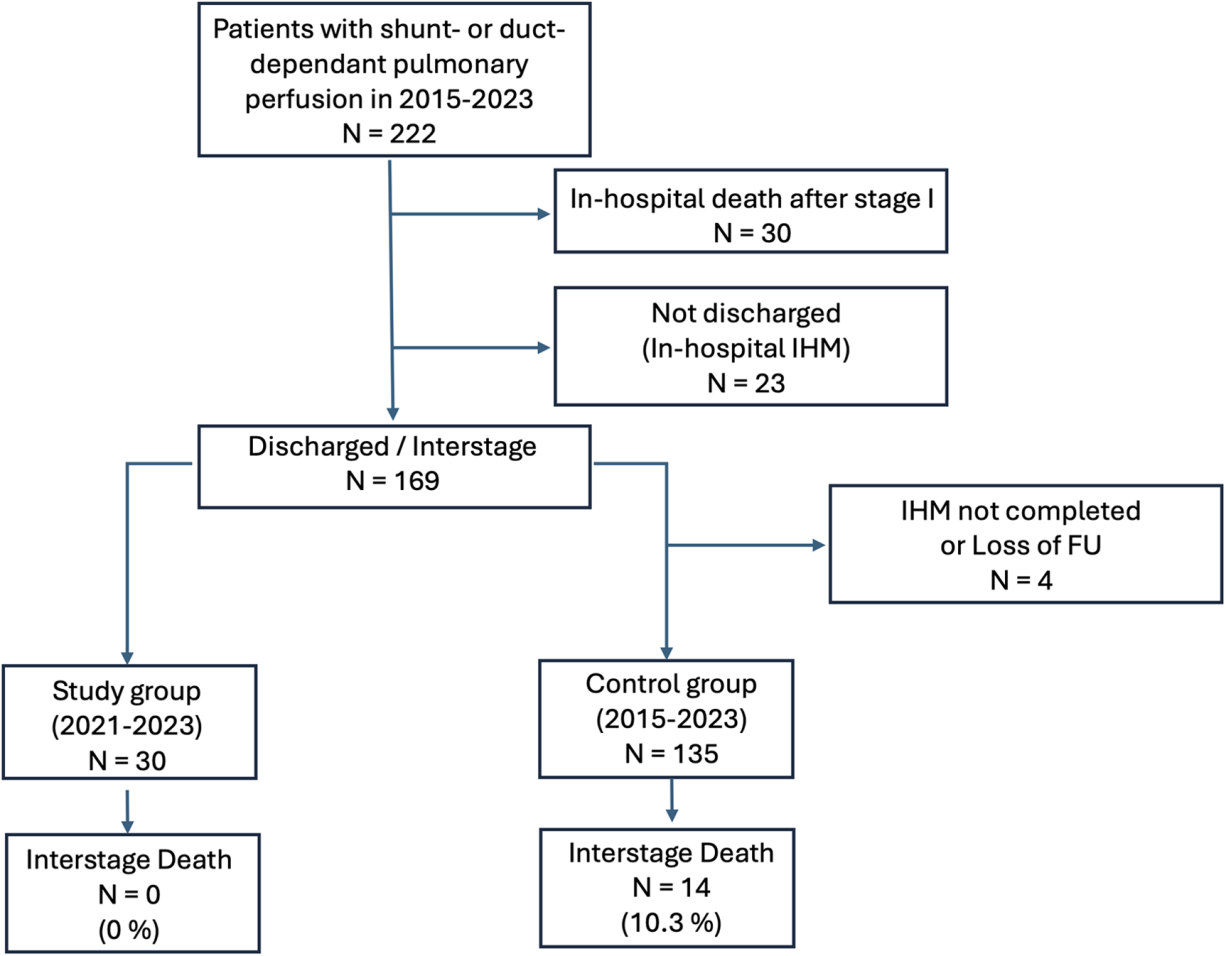
Flowchart of patients treated with shunt- or duct-dependent pulmonary perfusion between 2015 and 2023 and their distribution into the study and control cohort.

To evaluate the effect of the application-based remote interstage monitoring on the primary outcome survival to stage II procedure, we compared the results with a control cohort of 192 patients, who underwent surgery within 5 years before the implementation of RPM and those who did not participate but were palliated during the study period (stage I palliation between January 2015 and October 2023). Between 2015 and 2023, we treated 222 patients with shunt- or duct-dependent pulmonary perfusion at our center (Figure 5). Thirty patients died during the postoperative course after the first procedure, and 23 high-risk patients were not discharged receiving in-hospital surveillance during the interstage period. A total of 169 patients were discharged after S1P to IHM, of which 30 patients were monitored with RPM in the study group and 135 were assigned to the control group without RPM as described previously. Six patients were excluded as they either did not yet complete IHM or there was a loss of follow-up. Table 4 outlines the demographics and outcome variables of the study and the control group. There were no statistically significant differences in sex, diagnoses, or surgical interventions between both groups. Notably, we had a slightly higher number of HLHS patients in our study cohort compared to the control group (50 vs. 37%). There were significantly more Sano shunts and significantly less mBTS in our study group compared to the control group (*P* < 0.001). In the study cohort, significantly more patients required ECMO therapy during the postoperative course after S1P. Patients in the control group were significantly older at S1P compared to the study patients. We did not find any significant differences in birth weight, body weight at discharge or second procedure, or weight for age *Z* score (WAZ) at the according time points between study and control patients. The frequency of unplanned hospital admissions was slightly higher in the study cohort where 63% of the patients required at least an unplanned hospital admission compared to 44% of the patients in the control group, even though the difference was not statistically different. Unplanned interventions and procedures were comparable between both groups. Interstage mortality was 0% in the remotely monitored study group. In the control group, 14 of 135 patients died during the interstage period (10.3%). Interstage mortality in HLHS patients was even higher with 16% in the control group. The differences in interstage mortality failed to reach statistical significance (*P* = 0.065). In order to estimate the effect of the possible confounding risk factors that were distributed differently in the study cohort in comparison to the control group, we performed cox regression analysis to better estimate the effect of RPM on interstage mortality. Results are summarized in Table 5. We did not find any significant impact of the shunt type (either Sano or mBTS) on mortality. We did find that the diagnosis HLHS, of which more cases were in the study cohort compared to control group, was strongly associated with interstage mortality [hazard ratio (HR) 5.162, 95% CI: 1.171–22.654].

**Table 4:** Demographics and clinical outcomes in the study group and an unmatched historical control group.

	Median (IQR), *N* (%)		
	Study group 2021–2024 (*N* = 30)	Control group 2015–2023 (*N* = 135)	*P*-value
Sex			
– Male	13 (43%)	66 (51%)	0.441
– Female	17 (57%)	69 (49%)	
Diagnoses			
– HLHS	15 (50%)	50 (37%)	0.189
Surgery before IHM (first procedure)			0.703
– Norwood	20 (67%)	76 (56%)	
– Shunt	8 (27%)	50 (33%)	
– PDA stenting	2 (7%)	13 (10%)	
– Starnes + shunt		2 (2%)	
Shunt type			**<0**.**001**
– Sano shunt	16 (53%)	16 (11.9%)	
– Modified Blalock–Taussig shunt (mBTS)	9 (30%)	85 (63%)	
– Central aortopulmonary shunt	3 (10%)	21 (16%)	
– PDA stent	2 (8%)	13 (10%)	
Prior procedures			
– PAB	6 (20%)	29 (22%)	
– PDA stent	0 (0%)	6 (4%)	
– PAB + PDA stent	1 (3%)	3 (2%)	
ECMO after stage I	3 (10%)	3 (2%)	**0**.**040**
Surgery after IHM (second procedure)			0.083
– Glenn/comprehensive stage II	27 (90%)	89 (66%)	
– Biventricular correction	3 (10%)	29 (21%)	
Age (day)			
– At first procedure	6 (3–16)	21 (7–44)	**0**.**002**
– At discharge	48 (33–69)	55 (35–90)	0.213
– At second procedure	163 (143–188)	179 (138–277)	0.307
Body weight (kg)			
– At birth	3.15 (2.83–3.4)	3.13 (2.67–3.5)	0.662
– At discharge	3.92 (3.63–4.35)	3.91 (3.5–4.34)	0.785
– At second procedure	6.40 (5.12–7.13)	6.1 (5.49–7.04)	0.961
Weight for age Z score (WAZ)			
– At birth	−0.64 (−1.25 to 0.34)	−0.52 (−1.31 to 0.19)	0.780
– At discharge	−1.45 (−2.00 to −0.80)	−1.31 (−2.05 to −0.8)	0.787
– At second procedure	−1.11 (−2.02 to −0.50)	−1.37 (−1.95 to −0.57)	0.739
Unplanned hospital admissions			
– Total	32	106	0.102
– Per patient	1 (0–2)	0 (0–1)	
– Patients with at least 1	19 (63%)	60 (44%)	0.066
Unplanned inteventions/procedures			
– Total	32	108	0.815
– Per patient	1 (0–1)	1 (0–1)	
– Patients with at least 1	17 (57%)	72 (53%)	0.771
Interstage death			
– All patients	0 (0%)	14 (10.3%)	0.065
– HLHS patients	0 (0%)	8 (16%)	0.098

*P* values that were significant (*P* < 0.05) were highlighted bold.

**Table 5:** Multivariable Cox regression analysis.

Variable	Hazard ratio (HR)	95% CI	*P*-value
HLHS	5.152	1.171–22.654	**0**.**030**
Sano shunt	0.418	0.054–3.208	0.401
Modified BT shunt (mBTS)	0.451	0.086–2.373	0.347

*P* values that were significant (*P* < 0.05) were highlighted bold.

### 3.6 Comparison to a propensity score-matched cohort

To further estimate the effect of remote IHM, we used propensity score matching (PSM) to create a set of 28 case–control-matched cases adjusted for sex, diagnosis, type of surgery at the first procedure, and shunt type (Table 6). Comparing the study group with the PSM control group found no differences in surgical procedures, body weight, or WAZ at any time point. Patients in the PSM control group were significantly older at the second procedure. Results on clinical outcomes of both groups are summarized in Table 7. In the study cohort, more patients (63%) required at least one unplanned hospital admission in comparison with 43% of the PSM control group. This effect was not statistically significant. Of the 28 PSM cases, 4 died during the interstage period resulting in a significantly higher interstage mortality of 14% compared to 0% in the study group (*P* = 0.032). The same effect was seen in HLHS patients, interstage mortality was significantly higher with 27% in the PSM control group compared to 0% in the study cohort (*P* = 0.032).

**Table 6:** Propensity score-matched (PSM) cohort with the adjusted baseline variables.

	Median (IQR), *N* (%)		
	Study cohort (*N* = 30)	PSM cohort (*N* = 28)	*P*-value
Sex			
– Male	13 (43%)	16 (57%)	0.293
– Female	17 (57%)	12 (43%)	
Diagnoses			0.943
– HLHS	15 (50%)	11 (39%)	
– AVSD	4 (13%)	5 (18%)	
– CCTGA	3 (10%)	3 (11%)	
– DORV	2 (7%)	3 (11%)	
– PA/IVS	2 (7%)	1 (4%)	
– PA/VSD	1 (3%)	2 (7%)	
– HLHC	1 (3%)	0 (0%)	
– Tricuspid atresia	1 (3%)	2 (7%)	
– Shone	1 (3%)	1 (4%)	
Surgery before IHM (first procedure)			0.890
– Norwood	20 (67%)	17 (60%)	
– Shunt	8 (27%)	9 (32%)	
– PDA stenting	2 (7%)	2 (7%)	
Shunt type			0.550
– Sano shunt	16 (53%)	15 (54%)	
– Modified Blalock–Taussig shunt (mBTS)	9 (30%)	5 (18%)	
– Central aortopulmonary shunt	3 (10%)	6 (21%)	
PDA stent	2 (8%)	2 (7%)	

**Table 7:** Demographics and clinical outcomes in the study group and the propensity score-matched (PSM) cohort.

	Median (IQR), *N* (%)		
	Study cohort (*N* = 30)	PSM cohort (*N* = 28)	*P*-value
Prior procedures			0.563
– PAB	6 (20%)	5 (18%)	
– PDA stent	0 (0%)	1 (4%)	
– PAB + PDA stent	1 (3%)	0 (0%)	
ECMO after stage I	3 (10%)	1 (4%)	0.334
Surgery after IHM (second procedure)			0.265
– Glenn/comprehensive stage II	27 (90%)	19 (68%)	
– Biventricular correction	3 (10%)	5 (11%)	
Age (day)			
– At first procedure	6 (3–16)	19 (4–62)	0.060
– At discharge	48 (33–69)	69 (40–97)	0.061
– At second procedure	163 (143–188)	209 (163–309)	**0**.**015**
Body weight (kg)			
– At birth	3.15 (2.83–3.4)	3.25 (2.69–3.50)	0.987
– At discharge	3.92 (3.63–4.35)	4.1 (3.78–4.7)	0.155
– At second procedure	6.40 (5.12–7.13)	6.53 (5.72–7.84)	0.254
Weight for age *Z* score (WAZ)			
– At birth	−0.64 (−1.25 to −0.34)	−0.60 (−1.55 to −0.01)	0.415
– At discharge	−1.45 (−2.00 to −0.80)	−1.38 (−2.03 to −0.77)	0.974
– At second procedure	−1.11 (−2.02 to −0.50)	−1.37 (−2.09 to−0.27)	0.986
Unplanned hospital admissions			
– Total	32	17	0.070
– Per patient	1 (0–2)	0 (0–1)	
– Patients with at least 1	19 (63%)	12 (43%)	0.118
Unplanned inteventions/procedures			
– Total	32	22	0.783
– Per patient	1 (0–1)	1 (0–1)	
– Patients with at least 1	17 (57%)	16 (57%)	0.971
Interstage death			
– All patients	0 (0%)	4 (14%)	**0**.**032**
– HLHS patients	0 (0%)	3 (27%)	**0**.**032**

*P* values that were significant (*P* < 0.05) were highlighted bold.

### 3.7 Acceptance of technology and patient adherence

**Figure 6:**
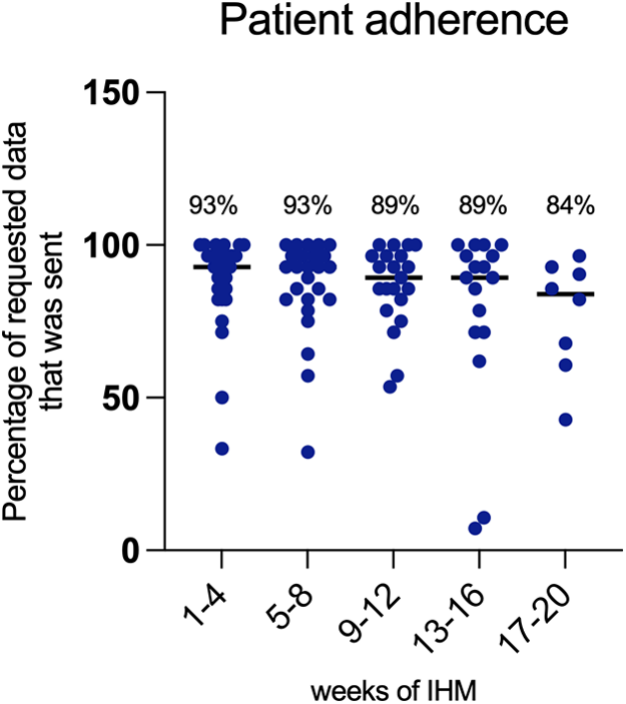
Patient adherence over the observation period expressed as the percentage of requested data that was sent over time.

The overall acceptance of application-based remote home monitoring was high with 96% of enrolled patients completing the observation period. Twenty-nine of the families that participated owned a smartphone or tablet, and only one family needed to be equipped with a tablet. Problems in using the application or in carrying out the home monitoring were mainly due to language issues, as we had a considerable proportion (27%) of non-native speakers among the parents. Patient adherence over time is illustrated in Figure 6 as the percentage of the requested data that were sent weekly. Overall, a median of 90.5% (IQR 82.1%–96.4%) of the requested data were sent weekly per patient. The percentage was 93% in the first 8 weeks and dropped slightly to 89% thereafter.

## 4 Discussion

In this clinical trial, we found that implementation of application-based remote interstage home monitoring for infants with single ventricle physiology and shunt- or duct-dependent lung perfusion was feasible. The acceptance of the technology and compliance of the families throughout the IHM period was very good as 96% finished the remote IHM by sending 90.5% of the requested data throughout the observation period. In our study cohort of 30 infants, we had no interstage deaths; therefore, interstage mortality was 0%. In comparison, interstage mortality in our control group was 10.3% overall and 16% for HLHS patients. Interstage mortality was even higher reaching statistical significance when cases of the control group were matched to the study cohort with regard to diagnosis, surgery, shunt type, and sex. There were statistically significantly more Sano shunts in the remote IHM study cohort in comparison to the control group. It is important to discuss the surgical approach to palliation as a factor influencing overall survival as differences in interstage mortality between patients with mBTS and patients with Sano shunt have been described previously, for instance in the single ventricle reconstruction trial (18% interstage mortality in mBTS patients vs. 6% in patients with sano shunt) (7). Other trials did not see significant differences in interstage mortality between Sano shunts and mBTS (12). In our results, the shunt type (Sano or mBTS) did not have a negative or positive impact on interstage mortality in our patient cohort assessed in a Cox regression model (HR for Sano shunt and mBTS was 0.418, 95% CI: 0.054–3.208, and 0.451, 95% CI: 0.086–2.373, respectively). The only risk factor with clear significance was the diagnosis of HLHS (HR 5.152, 95% CI: 1.171–22.654). Interestingly, even though the frequency of HLHS patients was higher in the study group (50%) in comparison to the control group 37%), interstage mortality was 0% in the study patients who were monitored with RPM. Another well-known risk factor for increased interstage mortality is ECMO therapy after the first procedure, which was significantly more used in the study cohort.

Furthermore, we could find a statistically significant higher interstage mortality in the control group, when cases were matched with PSM for diagnosis, shunt type, surgery, and sex. Reported interstage mortality in recent trials is between 8% and 12% in recently published studies (7, 12). The currently available data on this issue remain inconclusive and need to be addressed in larger study populations, as our patient cohort was rather small with only 30 patients. Nevertheless, we could find clear indicators for a positive effect of remote IHM on interstage mortality which was 0% in our small study cohort that contained a significant number of high-risk patients (such as HLHS patients and patients after ECMO therapy). Overall, the remote IHM program is a complex care system that most importantly facilitates communication between caregivers and the IHM health team in the hospital. The threshold to contact the IHM health team seemed to be lower due to the daily contact and the possibility to communicate through the application (this was a subjective impression by the IHM health care providers and not scientifically analyzed). In our opinion, the thorough training of the caregivers empowering them to carry out the monitoring independently and to be alert about red flag symptoms, combined with the improved digital data transfer and communication, was the decisive key factor in improving mortality.

The goal of IHM programs is the early identification of imbalances in systemic to pulmonary circulation and shunt- or duct-stent stenosis as well as the supervision and monitoring of heart failure treatment. Accordingly, higher readmission rates for infants who were monitored in designated IHM programs in comparison with those who were not have been reported. We observed a high incidence of unintended hospital readmissions which were necessary in 63% of all patients during the entire IHM period compared to 44% in the control group and 43% in the matched control group. The most common indications for hospital admissions were either cyanosis or infection in most of the cases. The higher rate of hospital admissions in the RPM study cohort could be an explanation for the reduction in interstage mortality as it indicates that infants were possibly earlier under clinical surveillance when red flag symptoms were present. Unintended interventions were necessary for 53%–57% of all patients, most of which were catheter-based interventions. The frequency did not differ between the study cohort and control patients. Infants admitted with cyanosis had significantly lower oxygen saturation in RPM data within 24 h before admission. Previous studies also reported frequent readmission rates and interventions (12). In our study cohort, shunt- or duct-stent stenosis followed by stenosis in the pulmonary arteries were the most frequent indications for catheter-based interventions. In our study cohort, recurrent coarctation occurred in two cases (6.7% of all patients). Recurrent coarctation was reported in up to 18%–27.3% of neonates after the Norwood procedure (13, 14). Our results and those of previous studies underline the importance of early recognition and treatment of hemodynamic-compromising anatomic lesions to reduce the rate of major cardiac events during the interstage period. The barrier to contacting the relevant specialists from the IHM team seems to be significantly lower through the application and the daily contact. This fosters a valuable relationship not typically established in a conventional setting. A direct visualization of the vital parameters in the application alerts the parents and the trend of relevant parameters such as oxygen saturation is monitored more precisely by medical professionals before a possible life-threatening desaturation occurs.

Besides the rapid identification and management of potentially serious and acute complications, RPM allows thorough address of non-acute issues such as optimizing somatic growth and adapting the medication according to the daily measured parameters to ensure optimal systemic to pulmonary circulation. Unnecessary visits and hospital admissions could be prevented. Interstage weight gain has become an important part of IHM management as it is a non-specific marker for cardiovascular health and a potentially modifiable risk factor for improving survival to stage II (4). Previous studies have demonstrated that a decline in weight for age *Z* scores during IHM results in worse surgical outcomes at stage II (15). Adequate calory intake targeting a normal weight gain is therefore crucial for IHM management and associated with improved outcomes at stage II (16). In our study cohort, the median weight gain was 20 g per day which refers to the lower range of normal weight gain in that age group. The weight for age *Z* score (WAZ) improved from −1.45 to −1.1 during IHM, even though the difference was not statistically significant. Even though WAZ was slightly better at the second procedure in the study cohort compared to both the control and the matched control group, we failed to significantly improve somatic growth with RPM. To date, no feeding modality has been proven to be superior to ensure adequate weight gain (16). In our study cohort, different feeding practices that were repeatedly evaluated and individually adapted could achieve adequate somatic growth. Those infants who failed to gain at least 15 g per day on average had a significantly lower weight for age *Z* score at admission to stage II surgery in our study cohort. It is striking that 50% of these infants were at least partially fed through a nasogastric tube with optimal calory intake in all cases through additional fortifier, which ultimately failed to result in optimal weight gain as they had the lowest WAZ compared to all other subgroups. This indicates that growth failure due to congestion is not always sufficiently treatable through adequate calory intake.

## 5 Conclusion

RPM offers a promising solution by using technology to remotely monitor and track the health status of children with single ventricle heart disease during the interstage period. Healthcare teams can closely monitor the child's condition and detect any concerning trends or deviations from the expected norms, enabling early intervention and adjustments to medications and feeding strategies. RPM facilitates communication between caregivers and healthcare teams and empowers parents and caregivers by actively involving them in their child's care and promoting shared decision-making. Application-based remote patient monitoring was feasible in this high-risk population and effective in preventing mortality. The implementation of RPM holds significant potential to improve outcomes, enhance patient safety, and reduce the burden on families. Despite numerous advantages, there are challenges to overcome when implementing RPM in in-home surveillance. Ensuring access to reliable internet connectivity and devices is essential. Moreover, healthcare systems need to establish a robust infrastructure for data collection, analysis, and secure transmission which needs substantial financial investments. To assess the predictability of adverse events by RPM data patterns, multicentered trials and machine learning models are required. This would add a novel layer of predictive analytics and help clinicians to anticipate adverse events earlier.

## 6 Limitations

The generalization of our results and statistical analysis is limited by the small size of our study cohort, which was not randomized, and the single-center approach. Due to the small number of patients and events, we did not reach statistical power to analyze the predictivity of RPM data on future major events. To further evaluate the impact of remote IHM programs on interstage mortality we emphasize the necessity of conducting larger, multicentered, randomized-controlled trials, given the rarity of the diseases. While the current design provides useful insights, it is subject to biases related to non-randomization and single-center recruitment. A larger multicentered RCT design would allow us to make stronger claims about the program's effect on mortality and adverse events.

## Data Availability

The raw data supporting the conclusions of this article will be made available by the authors, without undue reservation.
